# Higher Frontal Cortex Angiotensin Type 2 Receptor‐Interacting Protein (ATIP) Levels Are Associated With a Lower Amyloid‐Beta Burden in Postmortem Brains of Older Adults With Alzheimer's Disease

**DOI:** 10.1111/acel.70686

**Published:** 2026-08-30

**Authors:** Caglar Cosarderelioglu, Simion Kreimer, Alma I. Plaza‐Rodriguez, Pablo A. Iglesias, C. Conover Talbot, Helmy M. Siragy, Ceereena Ubaida‐Mohien, Francine Grodstein, Luigi Ferrucci, David A. Bennett, Jeremy Walston, Peter Abadir

**Affiliations:** ^1^ Division of Geriatric Medicine and Gerontology Johns Hopkins University School of Medicine Baltimore Maryland USA; ^2^ The Mass Spectrometry and Proteomics Facility The Johns Hopkins University School of Medicine Baltimore Maryland USA; ^3^ Krieger School of Arts and Sciences Johns Hopkins University Baltimore Maryland USA; ^4^ Department of Electrical and Computer Engineering, Whiting School of Engineering Johns Hopkins University Baltimore Maryland USA; ^5^ Institute for Basic Biomedical Sciences Johns Hopkins University School of Medicine Baltimore Maryland USA; ^6^ Department of Medicine, Division of Endocrinology and Metabolism University of Virginia Charlottesville Virginia USA; ^7^ National Institute on Aging, National Institutes of Health Baltimore Maryland USA; ^8^ Rush Alzheimer's Disease Center Rush University Medical Center Chicago Illinois USA

**Keywords:** Alzheimer's disease, angiotensin, angiotensin receptor blocker, AT_2_R‐binding protein (ATBP), AT_2_R‐interacting protein (ATIP), TOMAHAQ

## Abstract

Alzheimer's disease (AD) is a complex neurodegenerative disorder characterized by amyloid‐β (Aβ) and tau accumulation. Dysregulation of the brain renin‐angiotensin system, particularly hyperactivation of the angiotensin II type‐1 receptor, contributes to AD pathogenesis. In contrast, activation of the angiotensin II type‐2 receptor (AT_2_R) has been linked to neuroprotection and reduced Aβ accumulation. However, the underlying mechanisms of AT_2_R‐related Aβ reduction and the role of AT_2_R‐interacting protein (ATIP), also known as AT_2_R‐binding protein, remain unclear. We aimed to explore the relationship between ATIP and Aβ and tau pathologies as well as brain AT_2_R protein levels in older adults with AD. Using TOMAHAQ, a method that enables precise examination of numerous peptides across various samples in a single mass spectrometry analysis, we identified a specific human tryptic peptide that enables ATIP quantification. We applied this method to postmortem frontal‐cortex samples to measure ATIP levels. Sixty individuals with AD were included, half of whom were users of angiotensin receptor blockers (ARBs). The ATIP peptide was quantifiable in 12 participants. Among these individuals, higher ATIP levels were associated with lower Aβ burden in the frontal‐cortex and across multiple brain regions. This association remained significant after adjustment for age and ARB use. In contrast, ATIP levels were not significantly associated with AT_2_R, which was quantified using TOMAHAQ. This suggests that the relationship between ATIP and Aβ burden may not depend on differences in AT_2_R abundance. Although causality cannot be established, these findings may suggest a potential protective role for ATIP in AD that warrants further investigation.

## Graphical abstract

Higher levels of angiotensin II type‐2 receptor‐interacting protein (ATIP), quantified by TOMAHAQ mass spectrometry, were associated with lower amyloid‐β burden in postmortem brains of older adults with Alzheimer's disease (AD). These findings suggest ATIP may play a protective role in AD pathogenesis, warranting further investigation.

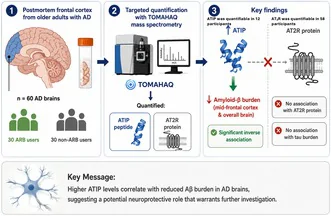

## 1 Introduction

Alzheimer's disease (AD) is a multifaceted, age‐related neurodegenerative disease characterized by progressive neuronal loss, tau pathology, increased amyloid‐β (Aβ), oxidative stress, and glial cell activation (Hardy and Selkoe 2002; McGeer and McGeer 2003, 2013). AD affects an estimated 7.2 million Americans aged 65 and older, and its prevalence is expected to double to 13.8 million by 2060, making it one of the most pressing public health challenges in the United States (Alzheimer's Association 2025). This growing burden is not only linked to high mortality and rising care costs but also to the emotional and physical toll on millions of unpaid caregivers and a strained dementia care workforce (Alzheimer's Association 2024). Despite the identification of several molecular processes, including oxidative stress, mitochondrial dysfunction, and chronic inflammation, the most significant aging‐related mechanisms associated with AD remain elusive.

Recent evidence suggests a link between AD and a dysregulated brain renin‐angiotensin system (bRAS), primarily due to the hyperactivation of the brain angiotensin II type‐1 receptor (AT_1_R), which can lead to inflammation, oxidative stress damage, mitochondrial decline, and impaired autophagy (Cosarderelioglu et al. 2020; Labandeira‐Garcia et al. 2017; Wright and Harding 2019; Cosarderelioglu et al. 2021). In the bRAS, angiotensin II (Ang II) mainly interacts with two subtypes of receptors: AT_1_R and Angiotensin II type‐2 receptor (AT_2_R). AT_2_R is a relatively understudied receptor subtype that canonically functions through nitric oxide release and is associated with vasodilation, neurite outgrowth, and anti‐inflammation. It is part of the protective arm of the renin‐angiotensin system along with the angiotensin IV/AT_4_R and angiotensin (1–7)/MAS receptor pathways, which also contribute to protective mechanisms within this system (Abadir et al. 2006; Horiuchi et al. 2010; McFall et al. 2020; Abadir et al. 2024). In vivo studies showed that the AT_2_R has protective effects against stroke, AD, and cognitive impairment (Rodrigues‐Ferreira et al. 2013). Furthermore, activation of AT_2_R is linked to reductions in both cortical and hippocampal amyloid‐dense core plaques (Royea et al. 2020). Despite these protective roles, the signaling pathways of this enigmatic receptor and its connection to Aβ and tau pathologies in AD are not yet fully understood (Karnik et al. 2015).

Initial interest in the protective properties of AT_2_R in the brain stemmed from clinical studies that demonstrated the superiority of angiotensin receptor blockers (ARBs) over other antihypertensive drugs in cognition (Yasar et al. 2013; Hajjar et al. 2020). By selectively blocking AT_1_R, ARBs may enhance the effects of bRAS via the AT_2_R axis, thus underscoring AT_2_R's potential protective role in AD (Thöne‐Reineke et al. 2006; Gebre et al. 2018). Despite recent advancements in understanding AT_2_R's structure (Asada et al. 2020), many important questions about AT_2_R remain unanswered, such as its activation and downstream signaling via different pathways (Sadybekov and Katritch 2020). In addition to Ang II, other angiotensin peptides have been identified as AT_2_R ligands, including compound 21, Ang III, Ang IV, and Ang‐(1–7), among others (Karnik et al. 2015; Bosnyak et al. 2011; Soda et al. 2020). Recently, a family of AT_2_R‐interacting proteins, known as ATIP proteins (ATIP1‐4), has also been discovered (Rodrigues‐Ferreira and Nahmias 2010). These proteins are encoded by a single gene, MTUS1 (microtubule‐associated tumor suppressor), through alternative exon splicing and alternative promoters (Bozgeyik et al. 2017). All ATIP proteins share a conserved C‐terminal amino‐acid sequence containing the AT_2_R binding site (Rodrigues‐Ferreira et al. 2013; Di Benedetto et al. 2006; Haykal et al. 2021). ATIP has been shown to interact with the AT_2_R, facilitating its localization to the cell membrane (McFall et al. 2020; Rodrigues‐Ferreira et al. 2013; Nouet et al. 2004). Furthermore, ATIP has been shown to mediate some AT_2_R functions (Soda et al. 2020), and dysfunctional interaction between ATIP and AT_2_R has been suggested as a contributing factor in certain AT_2_R‐related diseases (Wruck et al. 2005). Notably, ATIP1 expression is highest in the central nervous system, while ATIP4 expression is primarily found in the central nervous system tissues, suggesting the significance of ATIP in the brain (Di Benedetto et al. 2006). The association of ATIP with the microtubule cytoskeleton, the role of AT_2_R in preserving cognitive function and reducing Aβ, and the role of the ATIP protein in AT_2_R function have led to the hypothesis that ATIP may be a key factor in the development of neurological disorders such as AD (Gallo‐Payet et al. 2011; Guimond and Gallo‐Payet 2012a). However, the molecular mechanisms by which AT_2_R signaling attenuates Aβ pathology remain poorly understood, and the role of its interacting partner, ATIP, in this process has not been fully explored. To our knowledge, no study has directly examined ATIP levels in the human AD brains or their relationship to hallmark pathologies.

In this study, we investigated the relationship between ATIP protein levels and AD pathologies, including Aβ and tau burden, in postmortem brains of older adults. This investigation was motivated by evidence of dysregulated brain renin–angiotensin system (bRAS) signaling in AD and reports that AT_2_R activation reduces Aβ accumulation (Cosarderelioglu et al. 2020; Cosarderelioglu et al. 2021; Kehoe 2018). ATIP levels were quantified using TOMAHAQ (triggered by offset, multiplexed, accurate mass, high resolution, and absolute quantification), a targeted mass spectrometry method that enables sensitive and specific quantification of multiple peptides across multiple samples in a single run. As a secondary aim, in light of accumulating epidemiological evidence suggesting a protective role of ARBs in AD, together with prior observations that AT_1_R blockade may shift signaling toward the potentially protective AT_2_R axis and our previous findings showing differences in brain angiotensin receptor levels between ARB users and non‐users, we examined whether ATIP‐related associations differed according to ARB use (Yasar et al. 2013; Cosarderelioglu, Nidadavolu, et al. 2023; Scotti et al. 2021; Ho et al. 2017). Finally, we assessed the relationship between ATIP and AT_2_R.  By clarifying the interplay between ATIP and AD pathologies, we aim to generate hypotheses regarding mechanisms of AT_2_R‐mediated neuroprotection and identify potential targets for future investigation.

## 2 Materials and Methods

### 2.1 Characteristics of the Participants

The postmortem frontal cortex brain samples were provided by the Rush Memory and Aging Project (MAP) (Bennett et al. 2018). The study was approved by the Institutional Review Board of Rush University Medical Center. All participants signed an informed consent, the Anatomic Gift Act, and a repository consent to allow their data to be repurposed. Subjects agreed to annual clinical evaluations and brain donation at the time of death. We included participants who were diagnosed with AD and had postmortem Aβ and tangle scores calculated by the Rush MAP study team. Participants who used angiotensin‐converting enzyme inhibitors (ACEIs) were excluded to avoid their potential confounding effects on renin–angiotensin system signaling. Age‐ and sex‐matched participants with AD (*n* = 60) who used ARBs (*n* = 30) and did not use ARBs (*n* = 30) were included in this study. Five were male (16.7%) in each group. The mean age was 90.11 (sd = 5.7) in the non‐ARB group and 90.20 (sd = 5.5) in the ARB group. Characteristics of participants are shown in Table 1. The diagnostic procedures for AD were previously reported (Bennett et al. 2006).

**TABLE 1:** Characteristics of participants.

	AD (no ARB) (*n* = 30)	AD + ARB (*n* = 30)
Age, mean (SD)	90.11 (5.7)	90.20 (5.5)
Sex, male, *n* (%)	5 (16.7)	5 (16.7)
BMI, median (IQR)	24.8 (23.4–29.0)	26.2 (24.6–30)
Hypertension, *n* (%)	25 (83.3)	24 (80)
PMI, h, median (IQR)	6.93 (4.25–10.58)	5.43 (4.4–13.2)

Abbreviations: AD, Alzheimer's disease; ARB, angiotensin receptor blocker; BMI, body mass index; PMI, postmortem interval.

### 2.2 Angiotensin II Type 2 Receptor‐Interacting Protein Quantification by TOMAHAQ

TOMAHAQ, a technique developed by the Gygi lab, integrates targeted peptide quantification with sample multiplexing via tandem mass tags (TMTs) (Erickson et al. 2017). The method enhances specificity by detecting “trigger peptides,” which are standard peptides labeled with an isotopically distinct yet structurally similar version of the same isobaric tag used for the samples (Rose et al. 2019). This approach allows for high‐throughput analysis, making TOMAHAQ suitable for studying tissues from large clinical cohorts. In this study, we applied TOMAHAQ to quantify ATIP. Details of the TOMAHAQ method can be found in Cosarderelioglu, Kreimer, et al. (2023).

#### 2.2.1 Reagents

High‐performance liquid chromatography (HPLC) grade acetonitrile and formic acid were procured from Thermo Fisher Scientific. Water was purified in‐house using a Millipore Milli‐Q filtration system. Triethylammonium bicarbonate (TEAB), iodoacetamide (IAA), dithiothreitol (DTT), acetone, trichloroacetic acid (TCA), and octyl‐β‐D‐glucopyranoside were purchased from Sigma‐Aldrich. Samples were digested using Trypsin/Lys‐C mix from Promega and labeled with TMT 10‐plex and TMT 0 (tandem mass tag, Thermo) reagents. The digested and labeled samples were desalted by solid‐phase extraction on an Oasis plate (Waters).

#### 2.2.2 Mass Spectrometry Analysis

Peptides (1 μL of each TMT set) were analyzed by reverse‐phase liquid chromatography interfaced with tandem mass spectrometry using an Easy‐nanoLC 1200 HPLC system (Thermofisher) interfaced with an Orbitrap Fusion Lumos Tribrid Mass Spectrometer (Thermofisher). TMT10plex/TMT0 labeled peptides in 2% acetonitrile/0.1% formic acid were loaded onto a C18 trap (S‐10 μM, 120 Å, 75 μm × 2 cm, YMC, Japan) and subsequently separated on a PicoFrit column (75 μm × 25 cm, 15u, ±1um tip, New Objective) in‐house packed with C18 phase (ReproSil‐Pur C18‐AQ, 3 μm, 120 Å, www.dr‐maisch.com) in a Phoenix S & T column heater at 50C using the following gradient conditions: sample loading in 100% A (2% acetonitrile 0.1% formic acid) with a rapid jump to 8% B (90% acetonitrile 0.1% formic acid), a linear ramp from 8% to 30% B over 90 min, linear ramp to 48% B over 15 min, ramp to 95% B over 5 min, 9 min hold at 95% B, and drop back to 0% B in 1 min. Eluting peptides were sprayed into the mass spectrometer through a 1 μm emitter tip (New Objective) at 2.6 kV.

TOMAHAQ analysis was performed using the method described by Erickson et al. (2017). In TOMAHAQ analysis, detection of the TMT0 labeled peptides triggers MS3 fragmentation of the endogenous receptor peptides to acquire more accurate reporter ion ratios by reducing isolation interference in reporter ion intensities. Briefly, precursor ions matching the TMT0 labeled trigger peptides were monitored in MS1 scans with ±10 ppm mass error tolerance. All matching ions above a 25,000‐intensity threshold were fragmented by CID at 34% collision energy using a 0.8 m/z isolation window. Detection of at least 6 predicted b or y ions (within 10 ppm mass error) was required in MS2 spectra at 15000 resolution to trigger the acquisition of MS2 fragmentation spectra of the corresponding target peptide. An initial MS2 scan of the target peptide ion was acquired at 60000 resolution (5e4 AGC and 900 ms IT) to identify the highest intensity fragment ions to be selected for quantitation. A second MS2 scan isolated the target peptide ion in a 0.4 m/z window and fragmented the ion by CID at 35% collision energy. Four TMT‐containing b or y ions in 2 m/z windows were synchronously (4 multi‐notch) isolated in the ion trap for further fragmentation by HCD at 65% collision energy in an MS3 scan at 60000 resolution with 5e5 AGC and 2000 ms IT. Reporter ion intensities were obtained from the MS3 spectra without compression from potential precursor ion isolation interference.

TOMAHAQ data were analyzed using the TOMAHAQ Companion app (Thermo) to enable manual evaluation of target peptide MS2 spectra and selection of quantitative MS3 scans.

### 2.3 Measurement of Protein Levels of Angiotensin II Type 2 Receptor

The protein levels of AT_2_R were measured by TOMAHAQ as described in our previous study (Cosarderelioglu, Kreimer, et al. 2023). The GNSTLATTSK peptide sequence of AT_2_R was identified and quantified.

### 2.4 Brain Histopathology and Measurement of Amyloid‐β and Tangle Scores by the Rush Alzheimer's Disease Center

Brain autopsies were performed, and Aβ and tangle scores were calculated by the Rush Alzheimer's Disease Center (RADC) using their established protocol, which combines molecularly specific immunohistochemistry and image analysis, as previously described (Schneider et al. 2009). Amyloid‐β protein was detected using molecularly specific immunohistochemistry and quantified through image analysis, with the value representing the percentage of cortical area occupied by Aβ. Aggregated Aβ proteins and paired helical filaments of tau tangles in brain regions were identified using the 10D5 antibody (1:300; courtesy of Elan Pharmaceuticals, South San Francisco, CA) and AT8 (1:2000, Thermo). Immunohistochemistry was conducted with diaminobenzidine as the reporter, supplemented by 2.5% nickel sulfate to enhance the contrast of the immunoreaction product. All sections were processed using a Biogenex automated immunohistochemical stainer (San Ramon, CA, USA) (Wilson et al. 2007). Aβ and tangle burden were calculated as the average percent density in eight relevant brain regions: entorhinal cortex, calcarine cortex, cingulate cortex, angular gyrus, inferior temporal gyrus, hippocampus, middle frontal gyrus (mf), and superior frontal cortex (Wilson et al. 2007). Overall Aβ scores for the entire brain were obtained using the mean values from all brain regions and then transformed to mean of square root (sqrt) by RADC. We analyzed associations between ATIP and AD pathology using both discrete brain region measures (mid‐frontal cortex) and averaged overall scores. Regional analysis allows direct comparison between ATIP levels and pathology in the same tissue source and may capture spatially distinct patterns of AD pathology, as Aβ and tau do not accumulate uniformly across the brain (Braak and Braak 1991). Overall score provides a more stable estimate of global pathology burden by reducing regional variability. For the overall Aβ score, the sqrt data were used in this study as the outcome variable, as recommended by RADC (RADC 2026).

### 2.5 Statistical Analysis

Correlation analysis was performed with Spearman correlation test using GraphPad Prism 10 (GraphPad Software, San Diego, CA, USA). The Student's *t*‐test was used for comparisons between the two groups after assessing the normality of the data using GraphPad Prism 10. SPSS 29 (IBM SPSS Statistics for Windows, version 29 (IBM Corp., Armonk, NY, USA)) was used for partial correlation analysis. Partial correlation analyses were performed to assess the relationship between ATIP peptide levels and Aβ load while controlling for potential confounders, including age and ARB use. Age was included as a covariate because it is the strongest known risk factor for Alzheimer's disease and is associated with both Aβ accumulation and alterations in renin–angiotensin system components; specifically, AT_1_R levels increase with age while AT_2_R levels decrease in the brain (Labandeira‐Garcia et al. 2017). ARB use was included as a covariate since ARBs block AT_1_R and may shift signaling toward the AT_2_R pathway, thereby indirectly affecting AT_2_R/ATIP signaling and Aβ metabolism. *p*‐value ≤ 0.05 was used as the threshold for statistical significance.

## 3 Results

We investigated the associations of ATIP with AT_2_R, Aβ pathology, and tau pathology in the brains of older adults with AD. The peptide sequence “ANLKNPQIMYLEQELESLK” of the ATIP protein was identified and quantified in 12 of the 60 participants, 75% of whom were women. Among these 12 participants, 5 were ARB users, and 7 were non‐ARB users.

### 3.1 Association of AT_2_R‐Interacting Protein With Alzheimer's Disease Brain Pathologies

**FIGURE 1:**
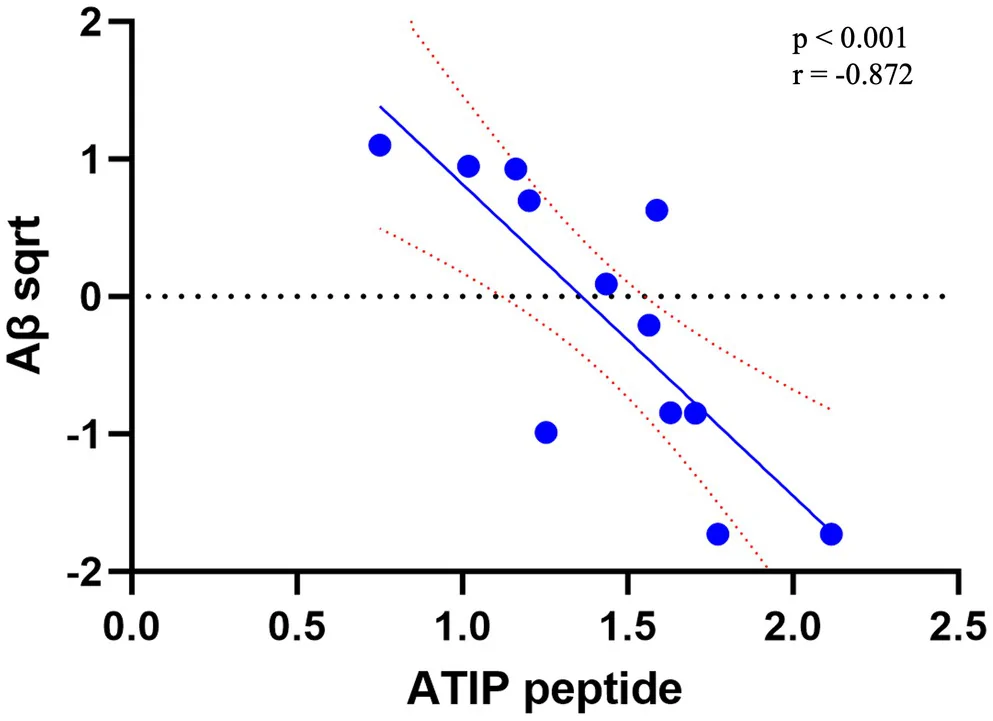
The correlation between Angiotensin II Type 2‐interacting protein and amyloid‐β score.

AD is characterized by the accumulation of Aβ plaques and neurofibrillary tangles (Srivastava et al. 2021). These pathological hallmarks are thought to contribute to synaptic dysfunction, neuronal loss, and cognitive decline. In our study, we analyzed the relationship between ATIP peptide levels and Aβ and tangle scores across eight brain regions, as well as the mean of these regions. Our results did not show any significant association between ATIP and tangle densities; however, we found a significant negative correlation between ANLKNPQIMYLEQELESLK peptide and Aβ load in the midfrontal gyrus (*p* = 0.001, *r* = −0.837), where ATIP peptides were quantified, as well as in all regions individually and with the overall Aβ sqrt score (*p* < 0.001, *r* = −0.872). When we performed a partial correlation analysis controlling for age and ARB use, the association remained significant for all regions including midfrontal and overall sqrt (Aβ mf *p* = 0.014, *r* = −0.743; Aβ sqrt *p* = 0.004, *r* = −0.814) (Table 2, Figure 1).

**TABLE 2:** Correlation between Angiotensin II Type 2‐interacting protein peptide and Amyloid‐β loads in all participants.

Brain region	Peptide sequence	*p*‐value—Spearman's correlation	Corr. Coeff. (*r*)	(Controlled for age and ARB use) – partial correlation	
				*p*	Corr. Coeff. (*r*)
Aβ mf	ANLKNPQIMYLEQELESLK	0.001	−0.837	0.014	−0.743
Aβ sqrt	ANLKNPQIMYLEQELESLK	< 0.001	−0.872	0.004	−0.814

Abbreviations: Aβ, amyloid‐β; ARB, angiotensin receptor blocker; mf, mid‐frontal; sqrt, square root.

### 3.2 Association of AT_2_R‐Interacting Proteins With Amyloid‐β in ARB Users and Non‐ARB Users

Next, to examine individual correlations in ARB users (5 participants) and non‐ARB users group (7 participants), we conducted correlation analysis between ATIP levels and Aβ load in the two groups.

In the non‐ARB group, there was a significant correlation between Aβ sqrt and ATIP levels (*p* = 0.012, *r* = −0.893). Additionally, in ARB users, there was a stronger significant negative correlation between ATIP and the overall Aβ sqrt score (*p* = 0.017, *r* < −0.999), demonstrating a more pronounced inverse correlation.

### 3.3 Association Between Protein Levels of ATIP and AT_2_R

To explore potential mechanisms linking ATIP to Aβ pathology, we examined the association between ATIP and AT_2_R protein levels. Since AT_2_R protein and ATIP have been implicated in various cellular processes, including vasodilation, anti‐inflammation, and anti‐fibrosis (Carey and Siragy 2003), understanding their interplay may provide valuable insights into the pathophysiology of AD.

AT_2_R protein levels were quantified by TOMAHAQ using the peptide sequence GNSTLATTSK in 58 participants. AT_2_R protein levels are presented in Table 3. We conducted correlation analyses between ATIP and AT_2_R in the combined cohort, ARB users, and non‐ARB users separately. However, no significant correlation was observed between ATIP and AT_2_R protein levels in any of the groups analyzed (ARB users: *r* = 0.000, *p* > 0.999; non‐ARB users: *r* = −0.036, *p* = 0.963; combined: *r* = −0.098, *p* = 0.766).

**TABLE 3:** Comparison of Angiotensin II Type 2‐interacting protein peptide levels, Angiotensin II Type 2 receptor protein levels, Amyloid‐β, and Tau pathology between Alzheimer's disease participants with and without Angiotensin receptor blocker treatment.

	AD (no ARB) (*n* = 7)	AD + ARB (*n* = 5)	*p*
ANLKNPQIMYLEQELESLK, mean (±SD)	1.375 (±0.284)	1.514 (±0.497)	0.549
GNSTLATTSK, mean (±SD)	2.48 ± (0.53)	2.38 ± (0.66)	0.779
Amyloid‐β sqrt (normalized), mean (±SD)	−0.051 (±1.103)	−0.319 (±1.057)	0.682
Tangle (normalized), mean (±SD)	−0.496 (±0.384)	−0.360 (±0.239)	0.468

Abbreviations: Aβ, amyloid‐β; AD, Alzheimer's disease; ARB, Angiotensin receptor blocker; sqrt, square root.

Additionally, AT_2_R protein levels did not differ significantly between ARB users (2.38 ± 0.66) and non‐ARB users (2.48 ± 0.53) (*p* = 0.779) (Table 3).

### 3.4 Comparison Between Participants With and Without Detectable ATIP

Next, we tested Aβ sqrt differences in ANLKNPQIMYLEQELESLK quantified (*n* = 12) and non‐quantified participants (*n* = 48) to understand if Aβ load has an effect on the quantification of the peptide. There was no statistically significant difference between the groups (*p* = 0.768), suggesting that detection status was not driven by Aβ load. Additionally, an intention‐to‐analyze approach assigning zero to non‐quantifiable samples and including all 60 participants showed no significant correlation between ATIP and Aβ sqrt (*r* = −0.124, *p* = 0.343), in contrast to the significant correlation observed among quantifiable samples.

### 3.5 Effect of Angiotensin Receptor Blocker Use on ATIP Levels

Finally, there was no significant difference in ATIP protein levels between ARB used (1.514 ± 0.497) and non‐ARB used (1.375 ± 0.284) groups (*p* = 0.549).

## 4 Discussion

In this study, we utilized mass spectrometry to quantify ATIP protein levels using a specific peptide sequence (ANLKNPQIMYLEQELESLK) and investigated the relationship between ATIP and brain pathologies, along with potential underlying mechanism, in older adults with AD who have either used or not used ARBs. We observed a negative correlation between ATIP levels and Aβ scores in older adults with AD, while no association was found between ATIP and AT_2_R levels. These findings contribute to a better understanding of the role of ATIP in AD and may have implications for future therapeutic strategies. While our findings are intriguing, they should be interpreted with caution due to the limitations of our study, including the small sample size and cross‐sectional design. These constraints prevent us from drawing definitive conclusions about causality or the broader implications of the observed relationships. Nonetheless, our findings underscore the need for further research to explore ATIP's function in the brain, including both AT_2_R‐dependent and ‐independent pathways.

The ATIP family, consisting of ATIP1, ATIP3, and ATIP4, is involved in regulating AT_2_R's trafficking, internalization, and/or activation (Rodrigues‐Ferreira et al. 2013; Haykal et al. 2021). These proteins are expressed in various brain areas and are implicated in several functions related to neuronal development and protection (Rodrigues‐Ferreira and Nahmias 2010; Bozgeyik et al. 2017; Di Benedetto et al. 2006). ATIP1 and ATIP3 have been recognized for their ability to inhibit the activation of extracellular signal‐regulated kinase (ERK) signaling, leading to the induction of apoptosis in cancer cells, and it is known that ATIP3 disrupts microtubule dynamics, resulting in prolonged mitosis (Bozgeyik et al. 2017). However, the precise role of ATIP isoforms in the brain and their involvement in neurodegenerative conditions like AD remain incompletely understood. To our knowledge, this is the first study to examine the association between ATIP and brain Aβ burden in humans.

The role of AT_2_Rs in neuronal development has long been a subject of interest, particularly due to their peak expression in the central nervous system during developmental stages, followed by a decline in adulthood. Previous studies have highlighted the involvement of AT_2_Rs in promoting neurite elongation, a crucial process in neuronal growth and connectivity. Activation of AT_2_Rs has been shown to induce neurite elongation in various cell types, suggesting a role in neural differentiation and development (Guimond and Gallo‐Payet 2012b; Horiuchi et al. 2012). Further elucidating the mechanisms underlying AT_2_R signaling, it has been demonstrated that upon activation, AT_2_Rs interact with ATIP, forming a complex with the tyrosine phosphatase SHP‐1. This complex translocates to the nucleus, where it activates methyl‐methanesulfonate‐sensitive 2 (MMS2), a ubiquitin‐conjugating enzyme variant, leading to neural differentiation and protection (Li et al. 2007). Additionally, it was shown that ATIP is involved in AT_2_R‐induced PPAR‐γ complex formation, and the crosstalk between AT_2_R and PPAR‐γ may be via Wnt10b/β‐catenin signaling (Shan et al. 2018). These interactions highlight the intricate crosstalk between AT_2_Rs and various signaling pathways involved in neural development and protection. In addition, the protective effects of ARBs against ischemic brain damage have been linked to PPAR‐γ activation and relative AT_2_R stimulation (Shan et al. 2018). Notably, ARB use also has been associated with a reduction in Aβ load in the brain (Nation et al. 2016; Ouk et al. 2021; Drews et al. 2019; Chou and Yeh 2014). Moreover, the therapeutic potential of AT_2_R agonists, such as C21, in reducing Aβ plaques in the brain has been demonstrated in mice, and it was suggested that these effects may be mediated, in part, by sustained microglial activation, implicating immune‐mediated mechanisms in the clearance of Aβ aggregates (Royea et al. 2020).

Despite the observed correlation between ATIP levels and Aβ scores, we did not detect a significant association between ATIP and AT_2_R expression. Several factors may account for this. First, AT_2_R is expressed at much lower levels in the adult brain compared to AT_1_R, and its abundance further decreases with age (Cosarderelioglu et al. 2020; Labandeira‐Garcia et al. 2017), making accurate quantification challenging in older adults. Second, AT_2_R protein expression may not always correspond with its activity. Third, ATIP may exert biological effects that are independent of AT_2_R signaling. A prior study using ATIP1 transgenic mice showed that the beneficial effects of ATIP on inflammation and oxidative stress persisted even in the presence of AT_2_R antagonists, suggesting ATIP‐specific, AT_2_R‐independent pathways (Jing et al. 2013).

This study has several limitations. The ATIP peptide could only be quantified in 12 out of 60 participants. This low detection rate reflects the technical challenges of quantifying low‐abundance proteins by targeted mass spectrometry. All 60 samples were processed identically using the TOMAHAQ assay, and the ATIP peptide (ANLKNPQIMYLEQELESLK) fell below the limit of quantification in the remaining 48 samples. Although the exact reason for this limited detection is unknown, it may be due to a combination of low endogenous peptide abundance in brain tissue and variability in tissue preservation across samples, postmortem interval, and individual biological variation in ATIP expression. To evaluate whether the subset with detectable ATIP differed systematically from the full cohort, we compared Aβ load between participants with and without quantifiable ATIP. No significant difference was observed. Nonetheless, the small number of participants with quantifiable ATIP limits statistical power and the generalizability of our findings, and results should be interpreted with caution. Future studies should consider strategies to improve ATIP detection and should include larger cohorts to validate these findings.

Moreover, we did not examine the roles of specific ATIP isoforms or related mechanisms which may mediate Aβ accumulation. These pathways warrant further investigation. We acknowledge that the use of postmortem brain tissue has inherent limitations, as the molecular changes observed may not fully represent the dynamic biological processes occurring in individuals with AD during life. In addition, factors such as tissue dissection variability, postmortem delay, and tissue pH may have contributed to measurement variability and should be considered when interpreting the results. Finally, although ARB exposure was examined as a clinically relevant variable, we were not able to account for differences among individual ARBs (Trigiani et al. 2018), including potential variation in blood–brain barrier penetrance and central effects. Additionally, another limitation of this study is the lack of an age‐matched cognitively normal control group. Consequently, we cannot determine whether ATIP levels differ between AD and cognitively normal aging, or whether the low detection rate is disease‐specific. Future studies should include cognitively normal controls to assess whether ATIP alterations are unique to AD. Importantly, our analysis is correlational and does not establish causality.

This study has several strengths as well. First, to our knowledge, this is the first study to quantify ATIP peptide levels in human postmortem brain tissue using targeted mass spectrometry (TOMAHAQ), providing direct relevance to human Alzheimer's disease biology that cannot be achieved by model systems alone. Second, our study design included age‐ and sex‐matched AD participants with and without ARB exposure, enabling us to explore whether ARB use influences ATIP levels and its relationship with Aβ pathology while minimizing potential confounding by demographic factors. Third, this study addresses a significant gap in the literature by examining ATIP, a relatively understudied protein in the context of AD, despite previous reports of its associations with AT_2_R, microtubule dynamics, and neurodevelopment. Finally, despite limitations including the small sample size and low detection rate, we observed a strong and statistically significant negative correlation between ATIP peptide levels and Aβ load that remained significant after adjustment for key covariates. Together, these strengths support the value of our exploratory findings and justify further mechanistic and translational studies to clarify ATIP's role in AD pathogenesis.

In summary, our exploratory study observed a negative correlation between the ATIP peptide sequence and Aβ scores in the brains of older adults with AD. These findings may suggest that an interplay between ATIP and related signaling pathways in the brain holds promise for understanding and potentially modulating neuroprotection and the pathogenesis of neurodegenerative diseases such as AD. Before any clinical or therapeutic relevance can be considered, future studies must first replicate these findings in larger and more diverse cohorts, include age‐matched cognitively normal controls, employ longitudinal designs to assess temporal relationships, and utilize functional and mechanistic models to determine whether ATIP plays a causal role in Aβ accumulation or AD progression. Further research is warranted to clarify the biological significance of ATIP in the brain and to evaluate whether it represents a potential target for future investigation in neurodegenerative diseases.

## 5 Conclusion

In this brief report, we investigated the relationship between ATIP peptide levels and brain pathology in older adults with AD. Our findings suggest a potential inverse correlation between ATIP levels and Aβ accumulation, indicating a possible role for ATIP in modulating AD pathology. Despite the small sample size, our results underscore the importance of further research into ATIP's role, both AT_2_R‐dependent and independent, in neurodegeneration. Future studies using larger cohorts and functional models are essential to validate these findings and explore ATIP as a potential therapeutic target in AD.

## Author Contributions

Caglar Cosarderelioglu, Simion Kreimer, Alma I. Plaza‐Rodriguez, Pablo A. Iglesias, C. Conover Talbot, Helmy M. Siragy, Ceereena Ubaida‐Mohien, Francine Grodstein, Luigi Ferrucci, David A. Bennett, Jeremy Walston, and Peter Abadir participated in study concept and design. Peter Abadir acted as the PI for the study. Simion Kreimer and C. Conover Talbot were responsible for the acquisition of the data. Caglar Cosarderelioglu, Simion Kreimer, C. Conover Talbot, Helmy M. Siragy, Ceereena Ubaida‐Mohien, Francine Grodstein, Luigi Ferrucci, David A. Bennett, Jeremy Walston, and Peter Abadir participated in the analysis and interpretation of the data. All authors participated in drafting the manuscript and approved the final version.

## Funding

This work was supported by the Bright Focus Foundation Research Award (P.A.); the Johns Hopkins University Claude D. Pepper Older Americans Independence Center, which is funded by the National Institute on Aging of the National Institutes of Health (NIH) under award number P30AG021334; and NIH grants R01AG046441 and K24AG088484 (P.A.); the Nathan W. and Margaret T. Shock Aging Research Foundation, Nathan Shock Scholar in Aging (P.A.).

## Ethics Statement

Postmortem frontal cortex brain samples came from participants in the Rush Memory and Aging Project. The study was approved by the Institutional Review Board of Rush University Medical Center. All participants signed an informed consent, Anatomic Gift Act, and a repository consent to allow their data to be repurposed.

## Conflicts of Interest

The authors declare no conflicts of interest.

## Data Availability

The data supporting this study's findings are not publicly available. However, the data can be provided upon reasonable request for specific research purposes.
